# Prevalence of Biologically vs Clinically Defined Alzheimer Spectrum Entities Using the National Institute on Aging–Alzheimer’s Association Research Framework

**DOI:** 10.1001/jamaneurol.2019.1971

**Published:** 2019-07-15

**Authors:** Clifford R. Jack, Terry M. Therneau, Stephen D. Weigand, Heather J. Wiste, David S. Knopman, Prashanthi Vemuri, Val J. Lowe, Michelle M. Mielke, Rosebud O. Roberts, Mary M. Machulda, Jonathan Graff-Radford, David T. Jones, Christopher G. Schwarz, Jeffrey L. Gunter, Matthew L. Senjem, Walter A. Rocca, Ronald C. Petersen

**Affiliations:** 1 Department of Radiology, Mayo Clinic, Rochester, Minnesota; 2 Department of Health Sciences Research, Mayo Clinic, Rochester, Minnesota; 3 Department of Neurology, Mayo Clinic, Rochester, Minnesota; 4 Department of Nuclear Medicine, Mayo Clinic, Rochester, Minnesota; 5 Department of Psychiatry and Psychology, Mayo Clinic, Rochester, Minnesota

## Abstract

**Importance:**

A National Institute on Aging–Alzheimer’s Association (NIA-AA) workgroup recently published a research framework in which Alzheimer disease is defined by neuropathologic or biomarker evidence of β-amyloid plaques and tau tangles and not by clinical symptoms.

**Objectives:**

To estimate the sex- and age-specific prevalence of 3 imaging biomarker–based definitions of the Alzheimer disease spectrum from the NIA-AA research framework and to compare these entities with clinically defined diagnostic entities commonly linked with Alzheimer disease.

**Design, Setting, and Participants:**

The Mayo Clinic Study of Aging (MCSA) is a population-based cohort study of cognitive aging in Olmsted County, Minnesota. The MCSA in-person participants (n = 4660) and passively ascertained (ie, through the medical record rather than in-person) individuals with dementia (n = 553) aged 60 to 89 years were included. Subsets underwent amyloid positron emission tomography (PET) (n = 1524) or both amyloid and tau PET (n = 576). Therefore, this study included 3 nested cohorts examined between November 29, 2004, and June 5, 2018. Data were analyzed between February 19, 2018, and March 26, 2019.

**Main Outcomes and Measures:**

The sex- and age-specific prevalence of the following 3 biologically defined diagnostic entities was estimated: Alzheimer continuum (abnormal amyloid regardless of tau status), Alzheimer pathologic change (abnormal amyloid but normal tau), and Alzheimer disease (abnormal amyloid and tau). These were compared with the prevalence of 3 clinically defined diagnostic groups (mild cognitive impairment or dementia, dementia, and clinically defined probable Alzheimer disease).

**Results:**

The median (interquartile range) age was 77 (72-83) years in the clinical cohort (n = 5213 participants), 77 (70-83) years in the amyloid PET cohort (n = 1524 participants), and 77 (69-83) years in the tau PET cohort (n = 576 participants). There were roughly equal numbers of women and men. The prevalence of all diagnostic entities (biological and clinical) increased rapidly with age, with the exception of Alzheimer pathologic change. The prevalence of biological Alzheimer disease was greater than clinically defined probable Alzheimer disease for women and men. Among women, these values were 10% (95% CI, 6%-14%) vs 1% (95% CI, 1%-1%) at age 70 years and 33% (95% CI, 25%-41%) vs 10% (95% CI, 9%-12%) at age 85 years (*P* < .001). Among men, these values were 9% (95% CI, 5%-12%) vs 1% (95% CI, 0%-1%) at age 70 years and 31% (95% CI, 24%-38%) vs 9% (95% CI, 8%-11%) at age 85 years (*P* < .001). The only notable difference by sex was a greater prevalence of the mild cognitive impairment or dementia clinical category among men than women.

**Conclusions and Relevance:**

Results of this study suggest that biologically defined Alzheimer disease is more prevalent than clinically defined probable Alzheimer disease at any age and is 3 times more prevalent at age 85 years among both women and men. This difference is mostly driven by asymptomatic individuals with biological Alzheimer disease. These findings illustrate the magnitude of the consequences on public health that potentially exist by intervening with disease-specific treatments to prevent symptom onset.

## Key Points

**Question:**

How does the prevalence of 3 imaging biomarker–based definitions of the Alzheimer disease spectrum from the National Institute on Aging–Alzheimer’s Association research framework compare with clinically defined diagnostic entities commonly linked with Alzheimer disease?

**Findings:**

Among a sample of 5213 individuals from Olmsted County, Minnesota, in this population-based cohort study, biologically defined Alzheimer disease is more prevalent than clinically diagnosed probable Alzheimer disease at any age and is 3 times more prevalent at age 85 years among both women and men.

**Meaning:**

Most patients with biologically defined Alzheimer disease are not symptomatic, which creates potential confusion around the definition of Alzheimer disease.

## Teaser

This population-based cohort study estimates the sex- and age-specific prevalence of 3 imaging biomarker–based definitions of the Alzheimer disease spectrum from the National Institute on Aging–Alzheimer’s Association research framework and compares these entities with clinically defined diagnostic entities commonly linked with Alzheimer disease.

## Introduction

Since 1984, a diagnosis of probable Alzheimer disease has been based on clinical findings of a progressive amnestic multidomain cognitive impairment culminating in dementia after other potential causes were excluded.^1,2,3^ Neuropathologic identification of β-amyloid plaques and tau tangles has always been required for a definite diagnosis of Alzheimer disease. Numerous studies have identified discrepancies between the clinical syndrome and the neuropathologic diagnosis of Alzheimer disease.^4^ Many individuals who meet neuropathologic criteria either do not have symptoms or have symptoms that differ from the classic amnestic presentation. National Institute on Aging–Alzheimer’s Association (NIA-AA) committees in 2011 and the International Work Group addressed this conundrum by adding biomarkers to clinical criteria to improve the specificity of the clinical diagnosis.^3,5,6,7,8^ These modified clinical diagnostic criteria cast Alzheimer disease as a clinical biomarker entity, but the diagnosis was not divorced from clinical impairment.^3,5,6,7,8^ The NIA-AA 2011 preclinical Alzheimer disease recommendations were an exception,^9^ as were the 2012 NIA-AA neuropathologic guidelines, which separated the neuropathologic definition of Alzheimer disease from the clinical syndrome.^10,11^

Building on the work above, a workgroup commissioned by the NIA-AA recently published a research framework^12^ defining Alzheimer disease biologically throughout its entire course. Alzheimer disease was defined either by neuropathologic examination or, in living persons, by positron emission tomography (PET) or biofluid biomarkers of the 2 hallmark diagnostic proteinopathies, namely, β-amyloid plaques and tau neurofibrillary tangles. The NIA-AA research framework harmonized the in vivo with the previously established neuropathologic^10,11^ definition of Alzheimer disease.

Epidemiologic studies estimating the prevalence of Alzheimer disease have typically used the clinical criteria by McKhann et al^1,3^ to define the condition. The new NIA-AA research framework^12^ leads to the following question: what is the prevalence of Alzheimer disease defined biologically using biomarkers compared with the prevalence using conventional definitions based on clinical symptoms? To address this question in the Mayo Clinic Study of Aging (MCSA) population, we estimated the sex- and age-specific prevalence of 3 imaging biomarker–based definitions of the Alzheimer disease spectrum from the NIA-AA research framework and compared these estimates with the prevalence of clinically defined diagnostic entities commonly linked with Alzheimer disease. Although biomarkers are now commonly used in aging and dementia research, most cohorts deeply phenotyped by biomarkers are clinic based and not population based. However, the MCSA is a population-based sample (ie, a random sample from a defined geographic area) with deep biomarker phenotyping.

## Methods

### NIA-AA Alzheimer Disease Spectrum Definitions

The NIA-AA research framework categorizes individuals with an abnormal amyloid but normal tau biomarker (A+T−) as having Alzheimer pathologic change.^12^ Both abnormal amyloid and tau biomarkers (A+T+) are required for a diagnosis of Alzheimer disease. The Alzheimer continuum is an umbrella definition encompassing any A+ individual, in whom a tau biomarker could be normal, abnormal, or unknown. These 3 biological definitions are referred to as the Alzheimer disease spectrum in this article. The A−T+ biomarker group is not relevant for the present work.

The NIA-AA research framework uses the AT(N) biomarker classification,^13^ where (N) refers to biomarkers of neurodegeneration or neuronal injury. However, (N) biomarkers are not specific for Alzheimer disease and thus are used in staging but cannot be used to define the disease, which is why the (N) is placed in parentheses. Therefore, (N) biomarkers are not relevant for the present work, which addresses the NIA-AA definitions of the Alzheimer continuum.

### Ascertainment, Enrollment, and Characterization

The MCSA is a population-based study of cognitive aging among a stratified random sample of Olmsted County, Minnesota, residents.^14^ Residents aged 30 to 89 years are enumerated using the medical records linkage system of the Rochester Epidemiology Project.^15^ From this sampling frame, individuals are randomly selected by 10-year age and sex strata such that women and men are equally represented. Because the prevalence of biomarker abnormalities is low below age 60 years,^16^ we included only individuals 60 years and older in the study. This study was approved by the Mayo Clinic and the Olmsted Medical Center Institutional Review Boards. All participants provided written informed consent at the time of enrollment.

Before 2015, the MCSA was focused on individuals who were cognitively unimpaired or had mild cognitive impairment (MCI). To assess eligibility, an electronic medical records screening procedure was used to passively identify individuals with dementia (and identify a possible etiology of dementia based on clinical presentation)^17^ for exclusion from the MCSA. Individuals determined to have a terminal illness were also excluded. Enumeration, stratified random sampling, and screening procedures are repeated to maintain roughly 3000 active participants who are evaluated approximately every 15 months.

A clinical diagnosis was determined for each in-person participant by a consensus committee composed of physicians, neuropsychologists, and study coordinators. The diagnosis of MCI was based on clinical judgment, including a history from the patient and informant.^18^ The diagnosis of dementia was based on *Diagnostic and Statistical Manual of Mental Disorders* (Fourth Edition) criteria.^19^ The diagnosis of clinically defined probable Alzheimer disease was based on the criteria by McKhann et al.^1,3^ Participants who did not meet criteria for MCI or dementia were deemed cognitively unimpaired.

**Figure 1.**
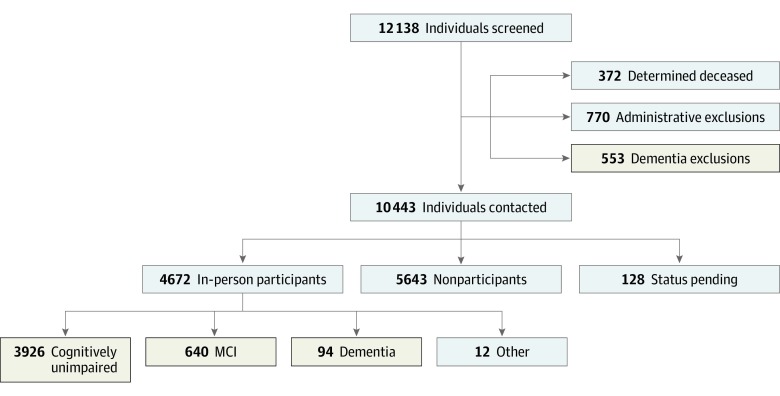
Flowchart Detailing the Study Design of the Mayo Clinic Study of Aging (MCSA) Among Individuals Aged 60 to 89 Years After enumeration of the Olmsted County, Minnesota, population, individuals’ medical records were screened for eligibility before being contacted to participate in the MCSA. Among the 770 administrative exclusions, 626 were unable to be contacted, 100 were terminally ill, and 44 were excluded for other reasons. Among the 5643 nonparticipants, 40 died after contact, 1847 participated by telephone only, and 3756 refused to participate. In-person participants were classified by cognitive status using a consensus diagnosis. The beige boxes represent the individuals included in the clinical cohort for this study, including cognitively unimpaired, MCI, and dementia groups from the MCSA plus individuals with dementia excluded from the MCSA. Twelve MCSA participants who could not be categorized as cognitively unimpaired, MCI, or dementia were excluded. MCI indicates mild cognitive impairment.

Figure 1 shows the screening and enrollment process of the MCSA for individuals aged 60 to 89 years. Individuals included in this study are the 4660 MCSA in-person participants with a diagnosis of cognitively unimpaired, MCI, or dementia (actively ascertained) and 553 passively ascertained individuals with dementia. Passive ascertainment was through review of the medical records in the medical records linkage system of individuals who had been randomly enumerated.

The MCSA participants without a medical contraindication were invited to participate in imaging studies. Amyloid PET was added to the MCSA in 2009 and tau PET in 2015; therefore, many more individuals have undergone amyloid PET alone than both amyloid and tau PET. For individuals with multiple imaging visits, the most recent visit with PET was used for this study.

Therefore, this study included 3 nested cohorts examined between November 29, 2004, and June 5, 2018. These comprised 5213 individuals in the clinical cohort (4660 in-person participants plus 553 passively ascertained with dementia), a subset of 1524 individuals in the cohort who underwent amyloid PET, and a subset of 576 individuals in the cohort who underwent both amyloid and tau PET.

### Imaging Studies

Amyloid PET was performed with Pittsburgh Compound B^20^ and tau PET with flortaucipir.^21,22^ Amyloid and tau PET standardized uptake value ratios (SUVRs) were formed by normalizing composite multiregion target regions of interest (ROIs) to the cerebellar crus gray matter.^23^ The amyloid PET target meta-ROI included the prefrontal, orbitofrontal, parietal, temporal, anterior and posterior cingulate, and the precuneus. The tau PET target meta-ROI used in the primary analysis included the amygdala, entorhinal cortex, fusiform, parahippocampal, and inferior temporal and middle temporal gyri.^23^ Cut points to determine normal vs abnormal studies were SUVR 1.48 (centiloid^24^ 22) for amyloid PET and SUVR 1.25 for tau PET using processing pipelines updated from previous work.^23^

### Statistical Analysis

Because the assessment of clinical status, amyloid PET, and tau PET occurred for nested cohorts of different sizes, the computation of prevalence was done in a staged fashion. We estimated the prevalence of clinical status (cognitively unimpaired, MCI, or dementia) among the clinical cohort, the prevalence of A+ by clinical group among the amyloid PET cohort, and the prevalence of T+ by clinical group and amyloid status among the tau PET cohort using multinomial and logistic regression. These prevalence estimates were then combined using basic probability rules to report the overall prevalence of the biomarker-defined entities among the Olmsted County population by sex and age. Complete details are provided in the eAppendix in the Supplement.

We also accounted for potential enrollment bias. Based on a recent MCSA analysis that identified sex, age, and education as the most important determinants of participation,^25^ we fit separate logistic regression models of participation (yes or no) in each stage of the process (initial enrollment, amyloid PET, and tau PET) using these 3 factors as predictors. These results were then used as inverse probability weights (IPWs) in the multinomial and logistic models explained above. Complete details are provided in the eAppendix in the Supplement.

We performed 2 types of sensitivity analyses to assess how different biomarker definitions change prevalence estimates of biologically defined Alzheimer spectrum entities. For each sensitivity analysis, we fit the same models described above but with different definitions of A+ or T+. First, we examined 3 additional ROIs for tau PET, namely, entorhinal cortex, inferior temporal, and lateral parietal ROIs that have been used in the field.^26,27,28,29^ Second, we varied the cut points for both abnormal amyloid and tau PET such that 10% more or 10% fewer cognitively unimpaired individuals would be classified as abnormal. Complete details are provided in the eAppendix in the Supplement. All *P* values were 2 sided, and *P* < .05 was considered statistically significant.

## Results

The median (interquartile range) age was 77 (72-83) years in the clinical cohort, 77 (70-83) years in the amyloid PET cohort, and 77 (69-83) years in the tau PET cohort. The Table lists the demographic characteristics of all individuals in this study (the clinical cohort) and the subsets with amyloid PET and tau PET. All individuals with tau PET also underwent amyloid PET. By design, the numbers of women and men were approximately equal in the overall sample, but those with MCI and those who were actively enrolled with dementia were more often male. Those who were passively identified as having dementia were less often male. Cognitively unimpaired individuals were younger, more educated, and had a lower frequency of *APOE* ε4 than those with MCI and dementia. The amyloid PET and tau PET subcohorts were comparable to the broader clinical cohort on age, education, the Short Test of Mental Status,^30^ and *APOE* ε4 distribution by clinical group (ie, there was no overall bias between those who were vs were not in the imaging subcohorts).

**Table.** Demographics of the 3 Nested Cohorts Examined in the Study^a^

Variable	Clinical Cohort				Amyloid PET Cohort			Tau PET Cohort		
	Cognitively Unimpaired	MCI	Dementia		Cognitively Unimpaired	MCI	Dementia	Cognitively Unimpaired	MCI	Dementia
			Actively Enrolled	Passively Ascertained						
No.	3926	640	94	553	1241	241	42	490	70	16
Sex, No. (%)										
Women	1989 (50.7)	280 (43.8)	38 (40.4)	308 (55.7)	585 (47.1)	99 (41.1)	14 (33.3)	227 (46.3)	33 (47.1)	6 (37.5)
Men	1937 (49.3)	360 (56.3)	56 (59.6)	245 (44.3)	656 (52.9)	142 (58.9)	28 (66.7)	263 (53.7)	37 (52.9)	10 (62.5)
Age, y										
Median (IQR) [range]	75 (71-81) [60-91]	81 (74-85) [60-91]	83 (80-87) [66-91]	84 (80-87) [61-91]	76 (69-82) [61-98]	82 (77-87) [61-97]	85 (81-87) [72-92]	76 (69-83) [61-98]	80 (74-85) [62-97]	85 (79-88) [76-90]
Education, y, No./total No. (%)^b^										
<12	210/3921 (5.4)	96/639 (15.0)	30/94 (31.9)	125/528 (23.7)	30/1241 (2.4)	24/240 (10.0)	4/42 (9.5)	9/490 (1.8)	6/69 (8.7)	1/16 (6.3)
12	1169/3921 (29.8)	250/639 (39.1)	30/94 (31.9)	186/528 (35.2)	321/1241 (25.9)	84/240 (35.0)	19/42 (45.2)	119/490 (24.3)	24/69 (34.8)	6/16 (37.5)
13-16	1733/3921 (44.2)	211/639 (33.0)	24/94 (25.5)	170/528 (32.2)	585/1241 (47.1)	94/240 (39.2)	14/42 (33.3)	249/490 (50.8)	28/69 (40.6)	7/16 (43.8)
>16	809/3921 (20.6)	82/639 (12.8)	10/94 (10.6)	47/528 (8.9)	305/1241 (24.6)	38/240 (15.8)	5/42 (11.9)	113/490 (23.1)	11/69 (15.9)	2/16 (12.5)
Short Test of Mental Status										
Median (IQR) [range]	35 (33-36) [19-38]	30 (28-32) [15-38]	24 (20-28) [4-33]	NA	36 (34-37) [24-38]	31 (28-33) [17-37]	26 (23-28) [14-32]	36 (35-37) [26-38]	32 (30-33) [22-37]	24 (22-28) [14-32]
*APOE ε4*, No./total No. (%)										
Noncarrier	2673/3628 (73.7)	390/569 (68.5)	44/83 (53.0)	NA	896/1215 (73.7)	136/224 (60.7)	19/40 (47.5)	333/465 (71.6)	34/53 (64.2)	6/14 (42.9)
Carrier	955/3628 (26.3)	179/569 (31.5)	39/83 (47.0)	NA	319/1215 (26.3)	88/224 (39.3)	21/40 (52.5)	132/465 (28.4)	19/53 (35.8)	8/14 (57.1)

Abbreviations: IQR, interquartile range; MCI, mild cognitive impairment; MCSA, Mayo Clinic Study of Aging; NA, not applicable; PET, positron emission tomography.

^a^ The clinical cohort includes MCSA individuals plus individuals with dementia excluded from the MCSA, categorized as passively ascertained. The amyloid PET cohort is a subset of the MCSA individuals in the clinical cohort, and the tau PET cohort is a subset of the amyloid PET cohort. The enrollment visit was used for the clinical cohort, and the most recent imaging visit was used for the amyloid PET and tau PET cohorts. We indicate the number of individuals with missing data for those variables with greater than 1% missing.

^b^ Based on information provided by Mayo Clinic patients for those who were passively ascertained.

**Figure 2.**
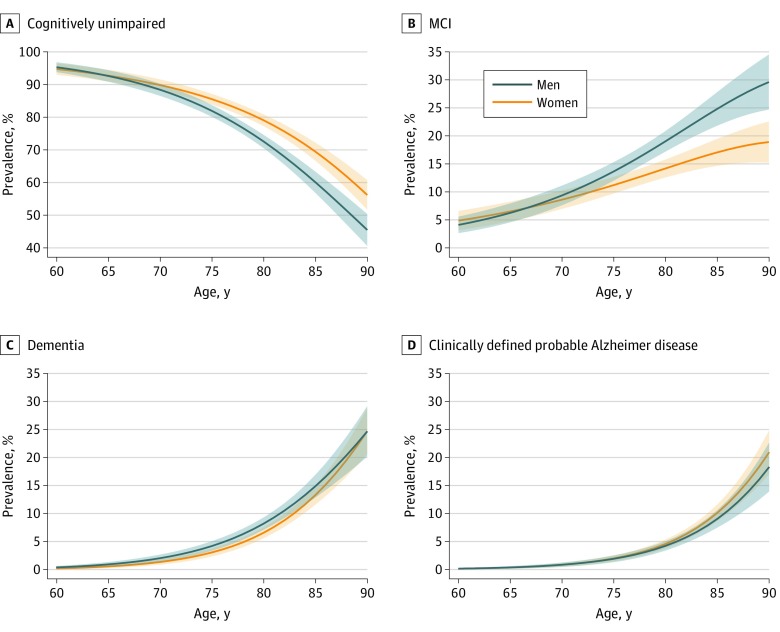
Prevalence of Clinically Defined Diagnoses A-D, Shown is the estimated prevalence of clinically defined diagnoses by sex and age with 95% CIs based on jackknife methods. Inverse probability weights were used to account for potential enrollment bias related to sex, age, and education. MCI indicates mild cognitive impairment.

Figure 2 shows the sex- and age-specific prevalence of each clinical diagnostic group. The prevalence of cognitively unimpaired status fell monotonically with age among women and men and was greater among women than men at 75 years and older (Figure 2A). The prevalence of MCI increased with age and was greater among men than women at 75 years and older (Figure 2B). The prevalence of dementia (Figure 2C) and clinically defined probable Alzheimer disease (Figure 2D) increased monotonically with age but was not significantly different for women vs men. eFigure 1 in the Supplement shows the prevalence of biologically defined NIA-AA Alzheimer spectrum diagnoses among clinical groups. The prevalence of Alzheimer continuum (A+), Alzheimer pathologic change (A+T−), and Alzheimer disease (A+T+) increased with age among cognitively unimpaired and MCI groups. We estimated an overall prevalence across all ages and both sexes among those with dementia due to limited sample sizes. Among those with dementia, the estimated prevalence of Alzheimer continuum (A+) was 77% (95% CI, 59%-95%), Alzheimer pathologic change (A+T−) was 8% (95% CI, 0%-21%), and Alzheimer disease (A+T+) was 68% (95% CI, 46%-91%).

**Figure 3.**
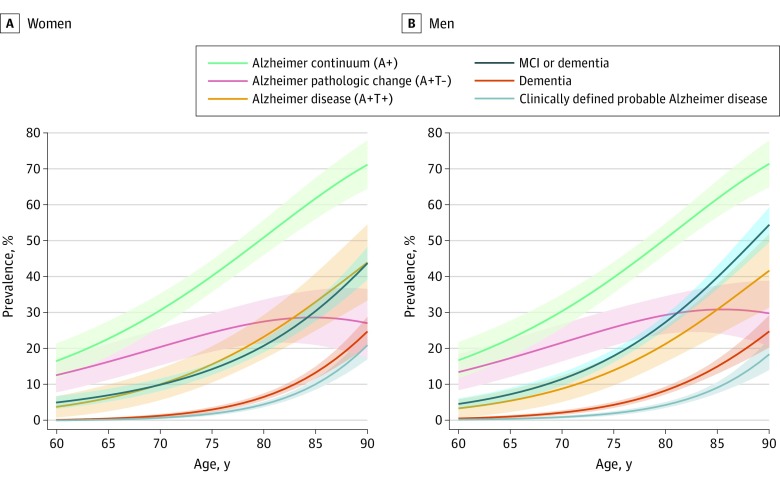
Prevalence of Biologically and Clinically Defined Diagnostic Entities A and B, Shown is the estimated prevalence of various entities by sex and age with 95% CIs based on jackknife methods. Inverse probability weights were used to account for potential enrollment bias related to sex, age, and education. Biologically defined Alzheimer disease spectrum entities are Alzheimer continuum (A+), Alzheimer pathologic change (A+T−), and Alzheimer disease (A+T+). Clinically defined syndromes are MCI or dementia, dementia, and clinically defined probable Alzheimer disease. MCI indicates mild cognitive impairment.

**Figure 4.**
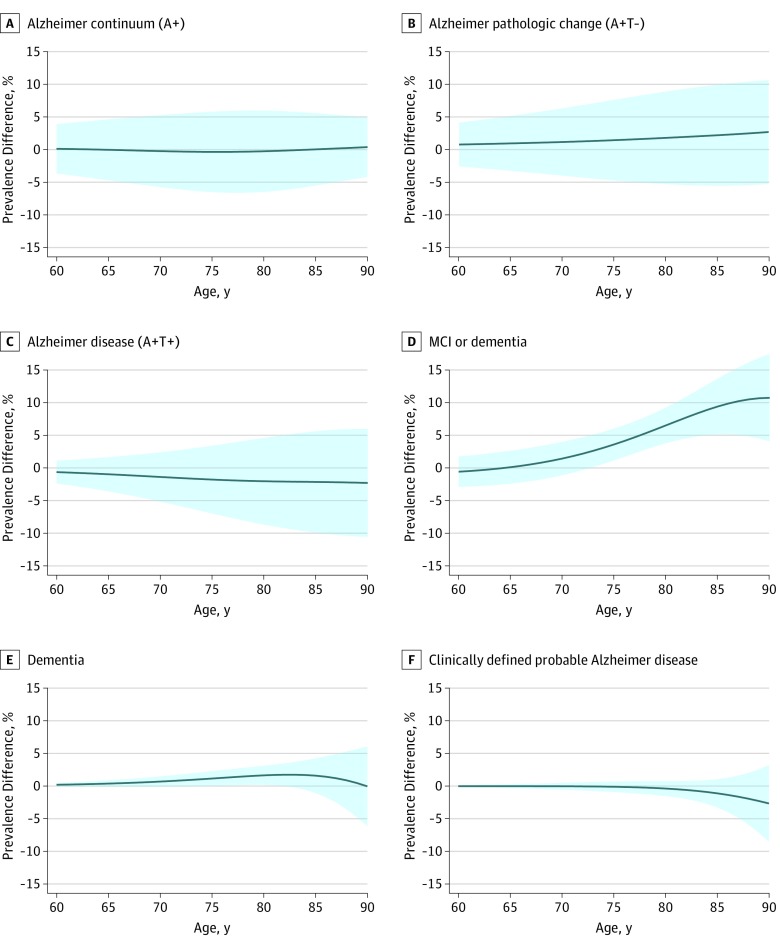
Sex Differences in the Prevalence of Biologically and Clinically Defined Diagnostic Entities The estimates shown in the graph are differences in the prevalence for men and women. Positive values indicate higher prevalence for men than women, negative values would indicate higher prevalence for women than men. Inverse probability weights were used to account for potential enrollment bias related to sex, age, and education. A-C, Biologically defined Alzheimer disease spectrum entities are Alzheimer continuum (A+), Alzheimer pathologic change (A+T−), and Alzheimer disease (A+T+). D-F, Clinically defined syndromes are MCI or dementia, dementia, and clinically defined probable Alzheimer disease. MCI indicates mild cognitive impairment.

Figure 3 shows our primary findings of the sex- and age-specific prevalence of biologically vs clinically defined diagnostic entities in Olmsted County. The prevalence of all diagnostic entities (biological and clinical) increased rapidly with age, with the exception of Alzheimer pathologic change (A+T−), which plateaued around age 80 years among women and men. Alzheimer continuum (A+) was the most frequent diagnostic entity in both men (*P* < .001) and women (*P* < .001): these values were 30% (95% CI, 26%-35%) in men and 31% (95% CI, 26%-35%) in women at age 70 years and 62% (95% CI, 57%-67%) in men and 62% (95% CI, 56%-67%) in women at age 85 years (Figure 3 and eTable in the Supplement). Alzheimer pathologic change (A+T−) was more common than biological Alzheimer disease (A+T+) in men (*P* = .05), but the difference was primarily seen at younger ages: these values were 22% (95% CI, 16%-27%) vs 9% (95% CI, 5%-12%) at age 70 years and 31% (95% CI, 24%-38%) vs 31% (95% CI, 24%-38%) at age 85 years. A similar pattern was seen in women, but the A+T− and A+T+ curves were not significantly different (*P* = .32): these values were 20% (95% CI, 15%-26%) vs 10% (95% CI, 6%-14%) at age 70 years and 29% (95% CI, 21%-36%) vs 33% (95% CI, 25%-41%) at age 85 years. At age 70 years in both women and men, the prevalence of clinically defined probable Alzheimer disease and the prevalence of dementia were very low: the respective values were 1% (95% CI, 0%-1%) and 2% (95% CI, 1%-3%) for men and 1% (95% CI, 1%-1%) and 1% (95% CI, 1%-2%) for women. At age 85 years in both women and men, the prevalence of biological Alzheimer disease (A+T+) was 3 times higher than that for clinically defined probable Alzheimer disease (9% [95% CI, 8%-11%] among men and 10% [95% CI, 9%-12%] among women), about twice as frequent as dementia (15% [95% CI, 13%-17%] among men and 13% [95% CI, 12%-15%] among women), and comparable to the frequency of the MCI or dementia group (40% [95% CI, 37%-43%] among men and 30% [95% CI, 28%-33%] among women). The only notable sex association across these entities was the greater prevalence of the MCI or dementia group among men than women (Figure 4).

A sensitivity analysis illustrated minor numeric differences^31^ in the prevalence of Alzheimer pathologic change (A+T−) and biological Alzheimer disease (A+T+) when the analyses were done with different tau PET reporter ROIs compared with the primary tau PET meta-ROI (eTable and eFigure 2 in the Supplement). The overall A+T+ prevalence curves were not different from the primary temporal meta-ROI among men or women using the entorhinal ROI or among women using the inferior temporal ROI or among men using the lateral parietal ROI. The overall A+T+ prevalence curves differed by a few percentage points from the primary temporal meta-ROI among men using the inferior temporal ROI and among women using the lateral parietal ROI (eTable and eFigure 2 in the Supplement). When the A and T cut points were changed to capture 10% more or 10% fewer individuals as abnormal, the prevalence of Alzheimer continuum (A+) generally increased or decreased by the expected 10 percentage points (eTable and eFigure 2 in the Supplement).

## Discussion

Estimates of the prevalence of clinical syndromes inform public health planners and public and private health agencies on present and near-future costs to society of supportive care.^32,33,34^ In contrast, the prevalence of biological definitions of the Alzheimer disease spectrum indicate the numbers of individuals by sex and age among a population who would be eligible for disease-modifying clinical trials that target specific aspects of Alzheimer disease biology. These data are necessary for planning disease-modifying clinical trials and ultimately for public health treatment strategies when disease-modifying treatments become available.^35,36,37^

Different methods of ascertainment and assessment and different operational definitions of clinically defined cognitive impairment syndromes have led to variable prevalence estimates of dementia.^32,38,39,40,41,42,43,44,45,46^ One reason for this variance is the use of a more conservative vs a more lenient definition of dementia^39,41^ that would include many individuals we have categorized as having MCI in this study. In addition, some studies^34,47,48,49^ have ascertained all-cause dementia, whereas other studies^50,51,52^ have ascertained clinically defined probable Alzheimer disease. For these reasons, we have included 3 different definitions of clinically defined impairment in this study. Defining cognitive impairment broadly as either MCI or dementia provides a most inclusive prevalence estimate, whereas restricting to clinically defined probable Alzheimer disease provides a most conservative estimate. The prevalences of MCI, dementia, and clinically defined probable Alzheimer disease reported herein are consistent with prior Olmsted County estimates^17,53^ but are not the primary focus of the present work. The main point we bring to the reader’s attention about prevalence estimates of the clinical diagnostic groups is that, through age 85 years, most women and men in the population are cognitively unimpaired (Figure 2). Consequently, the prevalence of biologically defined Alzheimer disease (A+T+) among a population (which sums individuals across the entire clinical spectrum [unimpaired, MCI, and dementia]) is heavily influenced by its prevalence among cognitively unimpaired individuals.

Compared with a biological definition of Alzheimer disease, a clinical definition will not include individuals who have the pathologic findings but do not have symptoms.^54^ In contrast, it will include symptomatic individuals who are clinically categorized as having probable Alzheimer disease in whom the etiology of dementia is disorders other than neuropathologically defined Alzheimer disease.^4,55^ The clinical definition will also leave persons with MCI in an ambiguous status. This highlights the need for more precise terminology around the concepts of dementia and Alzheimer disease. For this reason, the NIA-AA research framework recommended Alzheimer clinical syndrome to describe clinical syndromes (both dementia and MCI) that are commonly linked with Alzheimer disease.

The major objectives of this study were to compare the prevalence of biological vs clinical definitions of various diagnostic entities that have been linked with Alzheimer disease. The prevalence of all biological and clinical diagnostic entities we studied increased rapidly with age, with the exception of Alzheimer pathologic change (A+T−). Figure 3 and the eTable in the Supplement summarize several different comparisons, but the most direct biological vs clinical diagnostic comparison is between biological Alzheimer disease (A+T+) and clinically defined probable Alzheimer disease. At age 85 years, the prevalence of biological Alzheimer disease (A+T+) is 3 times higher than the prevalence of clinically defined probable Alzheimer disease (eTable in the Supplement).

A biological definition leads to an increase in the apparent prevalence of Alzheimer disease compared with a syndromal definition. This is not surprising; the same is true for any other disease (eg, cancer, diabetes, etc) in which tests can detect disease in both symptomatic and asymptomatic individuals. Even though there are no therapies proven to alter clinical outcomes, our data illustrate that a significant opportunity exists to influence public health by intervention in the preclinical phase of the disease if that proves to be efficacious.^56,57,58^ As other late-life diseases become better controlled, there is an imperative to delay or prevent symptoms due to Alzheimer disease; otherwise, those gains in life expectancy will be transformed into longer life with dementia. Intervention to prevent symptom onset was explicitly identified as a major public health objective in the National Plan to Address Alzheimer’s Disease.^36^

Many recent Alzheimer disease trials focus on anti-amyloid interventions and hence require evidence of amyloidosis for inclusion.^37,59,60,61^ However, as trials become increasingly sophisticated in targeting specific Alzheimer disease pathogenic pathways, information about both amyloid and tau status will be needed. For some interventions, the appropriate inclusion criterion will be Alzheimer pathologic change (A+T−), whereas for others it will be biologically defined Alzheimer disease (A+T+). Data provided in this article are useful for planning future clinical trials and ultimately for implementation of mechanism-specific interventions in pursuit of personalized medicine.

The only major sex association with the prevalence of the biological and clinical diagnostic groups was the higher prevalence of the MCI or dementia group among men (Figure 3 and Figure 4). As shown in Figure 2B, this is driven by a greater prevalence of MCI among men vs women, which has been demonstrated previously.^53^

Target and reference regions similar to those we used for amyloid PET are widely used throughout the Alzheimer imaging research community, and the approximate cut point we used, centiloid 22, has support in the literature.^62,63,64,65,66,67,68,69,70,71,72,73^ Because it is a newer modality, less agreement exists on optimal locations in the brain to measure ligand uptake or on cut points for tau PET.^22,26,29,74,75,76,77,78,79,80,81^ In sensitivity analyses, we found only minor numeric differences^31^ in the prevalence of biological Alzheimer disease (A+T+) when the analyses were done with different tau PET reporter ROIs compared with the primary tau PET meta-ROI (eTable and eFigure 2 in the Supplement). Therefore, our prevalence estimates seem generalizable and should not vary notably if different tau PET reporter ROIs were used.

### Strengths and Limitations

Other investigators^35,82,83,84,85^ and our group^16,25,86,87^ have estimated the prevalence of various Alzheimer biomarkers among clinically defined groups. However, a strength of the present study is that both biological and clinical diagnostic group prevalence estimates were ascertained among the same large population-based cohort, supporting straightforward biological vs clinical prevalence comparisons. Also, our T biomarker was tau PET, which is a new imaging modality whose importance is now being recognized.^21,22,26,29,58,74,88,89,90^

Another strength of our study is that we used a combination of active and passive surveillance for dementia case finding,^15^ which enabled us to capture the full spectrum of dementia diagnoses. Active surveillance will tend to detect mild cases that may not have been clinically recognized yet and thus have not appeared in the medical record. Passive surveillance will tend to detect more severe cases that have been recognized clinically and appear in the medical record. Although the prevalence of dementia is typically unknown among individuals who refuse participation in research studies, passive surveillance through the medical records linkage system^15^ enabled the identification of dementia among those who were randomly enumerated but did not actively participate in the study.

Our study had some limitations. We had fewer PET scans than desired in individuals with dementia. However, our estimates of A+T− and A+T+ in the population as a whole are precise because individuals with dementia make up a comparatively small proportion of the population. Other limitations are that the race/ethnicity of Olmsted County is predominantly white and the study setting is limited to the upper Midwest. Clinical diagnoses of MCI or dementia may be influenced by racial/ethnic and sociodemographic factors that are not generalizable to other populations.

A final limitation is that we were not able to perform biomarker studies in people with severe dementia. Individuals in nursing homes, hospice care, and other such settings cannot realistically participate in imaging research. This limitation is not unique to this study and will be found in any imaging or cerebrospinal fluid–based biomarker study.

## Conclusions

Biologically defined Alzheimer disease and clinically defined probable Alzheimer disease are not synonymous, which creates potential confusion around the definition of the term *Alzheimer disease*. The term *Alzheimer clinical syndrome* provides more precise terminology distinguishing between biological Alzheimer disease and the associated clinical syndrome.

## Notes

Supplement. **eAppendix.** Supplemental Appendix **eFigure 1.** Prevalence of Biologically Defined Alzheimer Disease Spectrum Entities by Clinical Group **eTable.** Prevalence Estimates (95% CI) by Sex at 2 Exemplar Ages (70 and 85) for the Primary Analysis and Several Sensitivity Analyses, Including Using Different Tau PET Reporter ROIs and Different Amyloid and Tau PET Cut Points **eFigure 2.** Prevalence of Biologically Defined Alzheimer Disease Spectrum Entities Using Different Tau PET Reporter ROIs and Different Amyloid and Tau PET Cut Points
