# Supplementary material for: Prevalence of Biologically vs Clinically Defined Alzheimer Spectrum Entities Using the National Institute on Aging–Alzheimer’s Association Research Framework
Source: JAMA Neurol. 2019 Jul 15;76(10):1174–83. doi: 10.1001/jamaneurol.2019.1971 (PMC6632154; doi:10.1001/jamaneurol.2019.1971)
Supplement: Supplement. — eAppendix. Supplemental Appendix eFigure 1. Prevalence of Biologically Defined Alzheimer Disease Spectrum Entities by Clinical Group eTable. Prevalence Estimates (95% CI) by Sex at 2 Exemplar Ages (70 and 85) for the Primary Analysis and Several Sensitivity Analyses, Including Using Different Tau PET Reporter ROIs and Different Amyloid and Tau PET Cut Points eFigure 2. Prevalence of Biologically Defined Alzheimer Disease Spectrum Entities Using Different Tau PET Reporter ROIs and Different Amyloid and Tau PET Cut Points [file jamaneurol-76-1174-s001.pdf]

Supplementary Online Content

Jack CR Jr, Therneau TM, Weigand SD, et al. Prevalence of biologically vs clinically defined Alzheimer spectrum entities using the National Institute on Aging–Alzheimer's Association research framework. *JAMA Neurol*. Published online July 15, 2019. doi:10.1001/jamaneurol.2019.1971

eAppendix. Supplemental Appendix

eFigure 1. Prevalence of Biologically Defined Alzheimer Disease Spectrum Entities by Clinical Group

eTable. Prevalence Estimates (95% CI) by Sex at 2 Exemplar Ages (70 and 85) for the Primary Analysis and Several Sensitivity Analyses, Including Using Different Tau PET Reporter ROIs and Different Amyloid and Tau PET Cut Points

eFigure 2. Prevalence of Biologically Defined Alzheimer Disease Spectrum Entities Using Different Tau PET Reporter ROIs and Different Amyloid and Tau PET Cut Points

eReferences.

This supplementary material has been provided by the authors to give readers additional information about their work.

© 2019 Jack CR Jr et al. *JAMA Neurology*.

eAppendix. Supplemental Appendix

Supplementary Statistical Methods

Expressing prevalence using rules of probability

Because the assessment of clinical status, amyloid PET, and tau PET occurred for nested cohorts of different size, the computation of prevalence was also done in a staged fashion using two rules of probability

(1) Pr(X and Y and Z) can be written as the product Pr(X) Pr(Y | X) Pr(Z | X, Y). This applies to any X, Y, Z; below they will be subsets of clinical status, amyloid PET status, and tau PET status, respectively.
(2) Pr(X) = Pr(C) Pr(X | C) + Pr(B) Pr(X | B) where C and B are mutually exclusive subsets of a population, e.g., male/female or A−/A+.

The overall prevalence of each clinical diagnosis (cognitively unimpaired [CU], MCI, dementia) is estimated using a three-category multinomial regression model fit within the entire clinical cohort consisting of 5213 individuals, 4660 of whom had an in-person MCSA visit and 553 of whom had passively ascertained dementia. Individuals with passively ascertained dementia were included in the model to enable us to estimate the prevalence of dementia in the entire population, which the MCSA was not originally designed to address. We note that multinomial regression is a generalization of logistic regression to the case where there are two or more unordered categorical outcomes.

The prevalence of A+, given clinical diagnosis, is based on a logistic regression model using the subset of MCSA participants with amyloid PET (n = 1524); and the prevalence of T+, given clinical diagnosis and amyloid status, is based on a logistic regression model using the subset of MCSA participants who had both amyloid and tau PET (n = 576) .

These prevalence estimates are then combined to get overall prevalence estimates of A+, A+T−, and A+T+ within a population, e.g.,

$$\Pr(A+) = \Pr(CU)\Pr(A+ \mid CU) + \Pr(MCI)\Pr(A+ \mid MCI) + \Pr(dementia)\Pr(A+ \mid dementia)$$

where Pr(A+) is the prevalence (probability) of A+, Pr(A+ | CU) is the prevalence of A+ given that the participant is cognitively unimpaired, etc. The formulas for prevalence of A+T− and A+T+ are similar:

$$\Pr(A+T-) = \Pr(CU)\Pr(A+ \mid CU)\Pr(T- \mid CU, A+) + \Pr(MCI)\Pr(A+ \mid MCI)\Pr(T- \mid MCI, A+) + \Pr(dementia)\Pr(A+ \mid dementia)\Pr(T- \mid dementia, A+)$$

and

$$\Pr(A+T+) = \Pr(CU)\Pr(A+ \mid CU)\Pr(T+ \mid CU, A+) + \Pr(MCI)\Pr(A+ \mid MCI)\Pr(T+ \mid MCI, A+) + \Pr(dementia)\Pr(A+ \mid dementia)\Pr(T+ \mid dementia, A+).$$

Using this approach, each term on the right hand side of the equation is able to use all study participants for whom relevant data was available.

For the multinomial model predicting clinical status, predictors were continuous age, sex, and the age×sex interaction. The model for A+ included continuous age and sex, fit separately for the CU and MCI groups. The model predicting T+ included additive continuous age, sex, clinical status, and amyloid status as predictors in the non-demented individuals. In the dementia group, due to small numbers of demented individuals with both amyloid PET and tau PET studies, the prevalence of A+ and T+ were each estimated overall rather than by age and sex.

Adjustment for potential enrollment bias

A second aspect of the modeling was to adjust for potential enrollment bias. A recent MCSA analysis by Roberts et al 2017 [1] that examined multiple factors identified age, sex, and education as the most important determinants of participation. Therefore, we used a logistic regression model to estimate the probability of in-person participation among all individuals that were contacted and invited to participate in the MCSA, minus those with participation status still pending (10443 – 128 = 10315). Predictors included age (ten-year age groups to match the MCSA sampling strata), sex, education (five level group: <12 years, 12 years, 13-15 years, 16 years, and >16 years), and all two-way interactions. The estimated probability of participation from this model was used to create inverse probability weights (IPW) for all MCSA in-person participants. These weights were used in the clinical status multinomial model to adjust for any participation bias. For this model, participants with passively identified dementia had a participation weight of 1 (since they had no opportunity to refuse).

© 2019 Jack CR Jr et al. *JAMA Neurology*.

Separate logistic regression models of participation in amyloid PET and tau PET were fit among the 10315 contacted individuals with the same predictors described above. These models were used to create IPWs for the A+ and T+ models, respectively.

To account for the IPW weights, jackknife estimates of variance were computed for all parameters. This was done by leaving out each participant in turn, re-fitting the clinical, A+, and T+ models, and then re-estimating the age and sex prevalence for each diagnostic entity to get standard errors.

For global comparisons of prevalence, we used the difference in mean height of each curve, from ages 60–90, along with a jackknife variance of that value.

Missing data
For this study, we categorized missing data as missingness due to non-participation in the MCSA versus missingness due to incomplete imaging. (No MCSA participants were missing age, sex, or clinical diagnosis.) A benefit to the analytic approach described above is that it accounts for both of these aspects of missing data. Inverse probability weighting addresses bias due to non-participation and bias due to incomplete imaging while our parameterization of prevalence allows us to maximize statistical efficiency by not requiring a complete-case analysis (i.e., an analysis that only uses individuals with both amyloid PET and tau PET).

© 2019 Jack CR Jr et al. *JAMA Neurology*.

eFigure 1. Prevalence of Biologically Defined Alzheimer Disease Spectrum Entities by Clinical Group

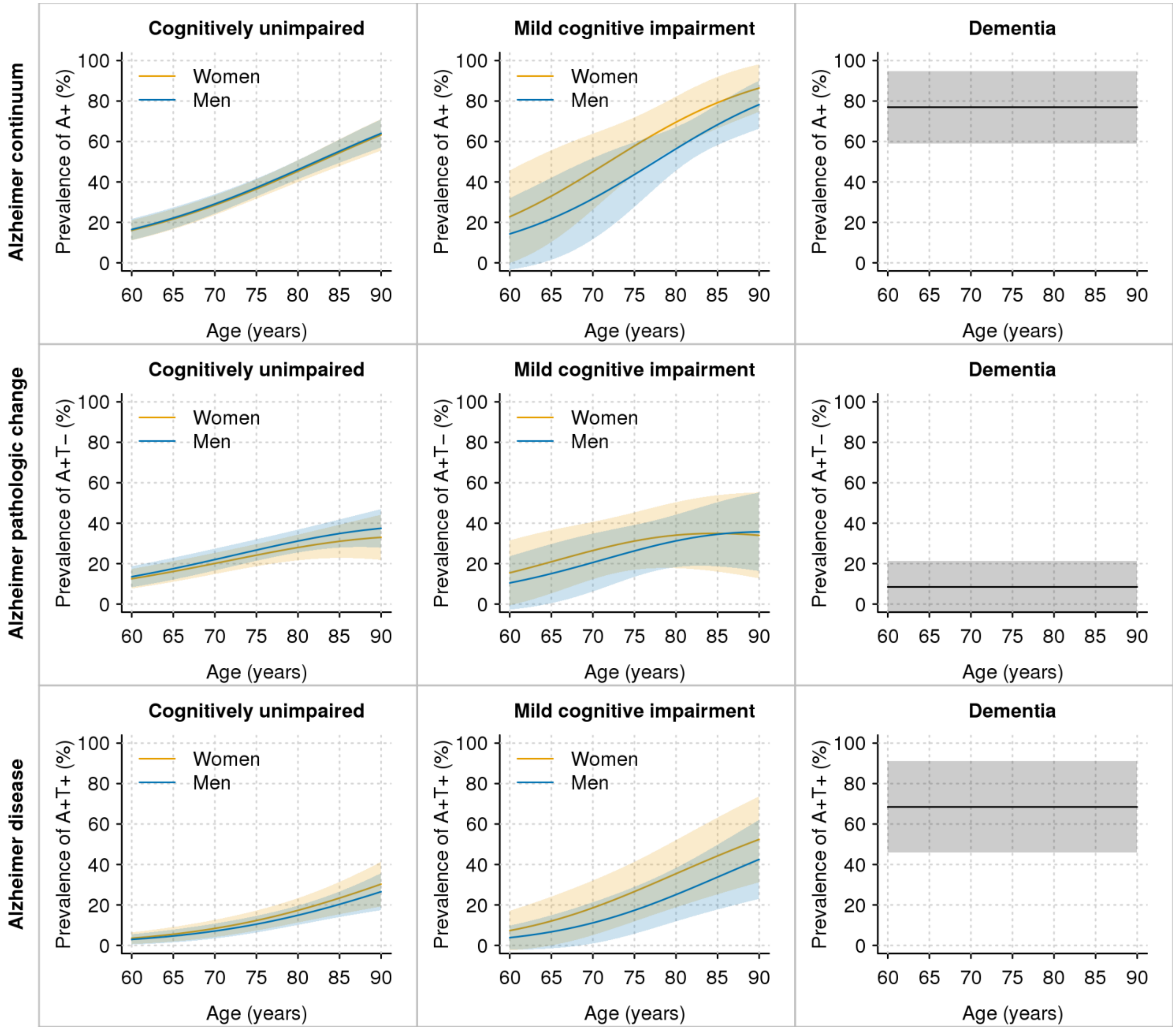

Estimated prevalence of Alzheimer continuum (A+), Alzheimer pathologic change (A+T−), and Alzheimer disease (A+T+) by age, sex, and clinical diagnosis with 95% confidence intervals based on jackknife methods. Inverse probability weights were used to account for potential participation bias related to age, sex, and education.

© 2019 Jack CR Jr et al. *JAMA Neurology*.

Supplementary Sensitivity Analyses

Our methodology and definitions of A+ and T+ have been described in detail in Jack et al 2017 [2]. The cut-points for these standard definitions using updated processing methods are ≥1.48 SUVR for A+ and ≥ 1.25 SUVR for T+. However, we recognize it is important to evaluate how sensitive our prevalence estimates are to different definitions of abnormal amyloid or abnormal tau. Therefore, we refit the models described in the main analysis using the following alternative definitions of A+ and T+:

Alternative definitions of A+:

1. Defining A+ using our standard region of interest (prefrontal, orbitofrontal, parietal, temporal, anterior and posterior cingulate, and precuneus) but raising the cut-point so that 10% fewer cognitively unimpaired individuals were identified as A+. The resulting, more conservative, cut-point was 1.58 SUVR.
2. Defining A+ using our standard region of interest but lowering the cut-point so that 10% more cognitively unimpaired individuals were identified as A+. The resulting, more lenient, cut-point was 1.43 SUVR.

Alternative definitions of T+:

1. Defining T+ based on tau PET levels in the entorhinal cortex (ERC). We used the same methodology described in our 2017 cut-points paper [2] to determine an ERC cut-point of 1.21 SUVR.
2. Defining T+ based on tau PET levels in the inferior temporal region. We used the same methodology described in our 2017 cut-points paper [2] to determine an inferior temporal cut-point of 1.28 SUVR.
3. Defining T+ based on tau PET levels in the lateral parietal region (supramarginal, angular, and inferior parietal). We used the same methodology described in our 2017 cut-points paper [2] to determine a lateral parietal cut-point of 1.23 SUVR.
4. Defining T+ using our standard region of interest (ERC, amygdala, parahippocampal gyrus, fusiform, inferior temporal, and middle temporal) but raising the cut-point so that 10% fewer cognitively unimpaired individuals were identified as T+. This more conservative cut-point was 1.29 SUVR.
5. Defining T+ using our standard region of interest but lowering the cut-point so that 10% more cognitively unimpaired individuals were identified as T+. This more lenient cut-point was 1.22 SUVR.

© 2019 Jack CR Jr et al. *JAMA Neurology*.

eTable. Prevalence Estimates (95% CI) by Sex at 2 Exemplar Ages (70 and 85) for the Primary Analysis and Several Sensitivity Analyses, Including Using Different Tau PET Reporter ROIs and Different Amyloid and Tau PET Cut Points

	**Women**		**Men**	
	**Age 70**	**Age 85**	**Age 70**	**Age 85**
**Definition**	**Prevalence (95% CI)**	**Prevalence (95% CI)**	**Prevalence (95% CI)**	**Prevalence (95% CI)**
**Primary analysis**				
MCI or dementia	10 (8, 12)	30 (28, 33)	11 (10, 13)	40 (37, 43)
Dementia	1 (1, 2)	13 (12, 15)	2 (1, 3)	15 (13, 17)
Clinically defined probable Alzheimer disease	1 (1, 1)	10 (9, 12)	1 (0, 1)	9 (8, 11)
Alzheimer continuum (A+)	31 (26, 35)	62 (56, 67)	30 (26, 35)	62 (57, 67)
Alzheimer pathologic change (A+T−)	20 (15, 26)	29 (21, 36)	22 (16, 27)	31 (24, 38)
Alzheimer disease (A+T+)	10 (6, 14)	33 (25, 41)	9 (5, 12)	31 (24, 38)
**Sensitivity analysis using tau in the entorhinal cortex (ERC) region as the reporter ROI**				
Alzheimer pathologic change (A+T−)	19 (14, 24)	26 (18, 33)	22 (17, 27)	31 (24, 38)
Alzheimer disease (A+T+)	12 (7, 16)	36 (28, 44)	8 (5, 12)	30 (23, 38)
**Sensitivity analysis using tau in the inferior temporal region as the reporter ROI**				
Alzheimer pathologic change (A+T−)	19 (14, 24)	26 (18, 33)	20 (15, 25)	28 (21, 35)
Alzheimer disease (A+T+)	12 (7, 16)	36 (28, 44)	10 (6, 14)	33 (26, 41)
**Sensitivity analysis using tau in the lateral parietal region as the reporter ROI**				
Alzheimer pathologic change (A+T−)	24 (19, 29)	37 (30, 45)	23 (19, 28)	37 (30, 43)
Alzheimer disease (A+T+)	7 (3, 10)	24 (17, 32)	7 (4, 10)	25 (18, 32)
**Sensitivity analysis varying tau and amyloid cut-points to classify 10% fewer individuals as abnormal***				
Alzheimer continuum (A+)	22 (18, 26)	55 (49, 60)	20 (16, 24)	53 (48, 58)
Alzheimer pathologic change (A+T−)	17 (13, 21)	30 (23, 37)	15 (11, 19)	28 (21, 34)
Alzheimer disease (A+T+)	6 (2, 9)	25 (17, 32)	5 (3, 8)	25 (18, 32)
**Sensitivity analysis varying tau and amyloid cut-points to classify 10% more individuals as abnormal***				
Alzheimer continuum (A+)	43 (38, 48)	68 (63, 73)	40 (35, 45)	66 (61, 71)
Alzheimer pathologic change (A+T−)	22 (16, 28)	24 (16, 31)	24 (18, 30)	27 (20, 34)
Alzheimer disease (A+T+)	21 (14, 27)	44 (36, 53)	16 (11, 21)	39 (32, 47)

* Among MCSA CU individuals, 482 (39%) were classified as A+ by the standard cut-point, 361 (29%) by the more conservative cut-point, and 598 (48%) by the more lenient cut-point; 127 (26%) were classified as T+ by the standard cut-point, 72 (15%) by the more conservative cut-point, and 188 (38%) by the more lenient cut-point.

Biologically defined Alzheimer disease spectrum entities are; Alzheimer continuum (A+), Alzheimer pathologic change (A+T−), and Alzheimer disease (A+T+). Clinically defined syndromes are MCI or dementia, dementia, and clinically defined probable Alzheimer disease.

© 2019 Jack CR Jr et al. *JAMA Neurology*.

eFigure 2. Prevalence of Biologically Defined Alzheimer Disease Spectrum Entities Using Different Tau PET Reporter ROIs and Different Amyloid and Tau PET Cut Points

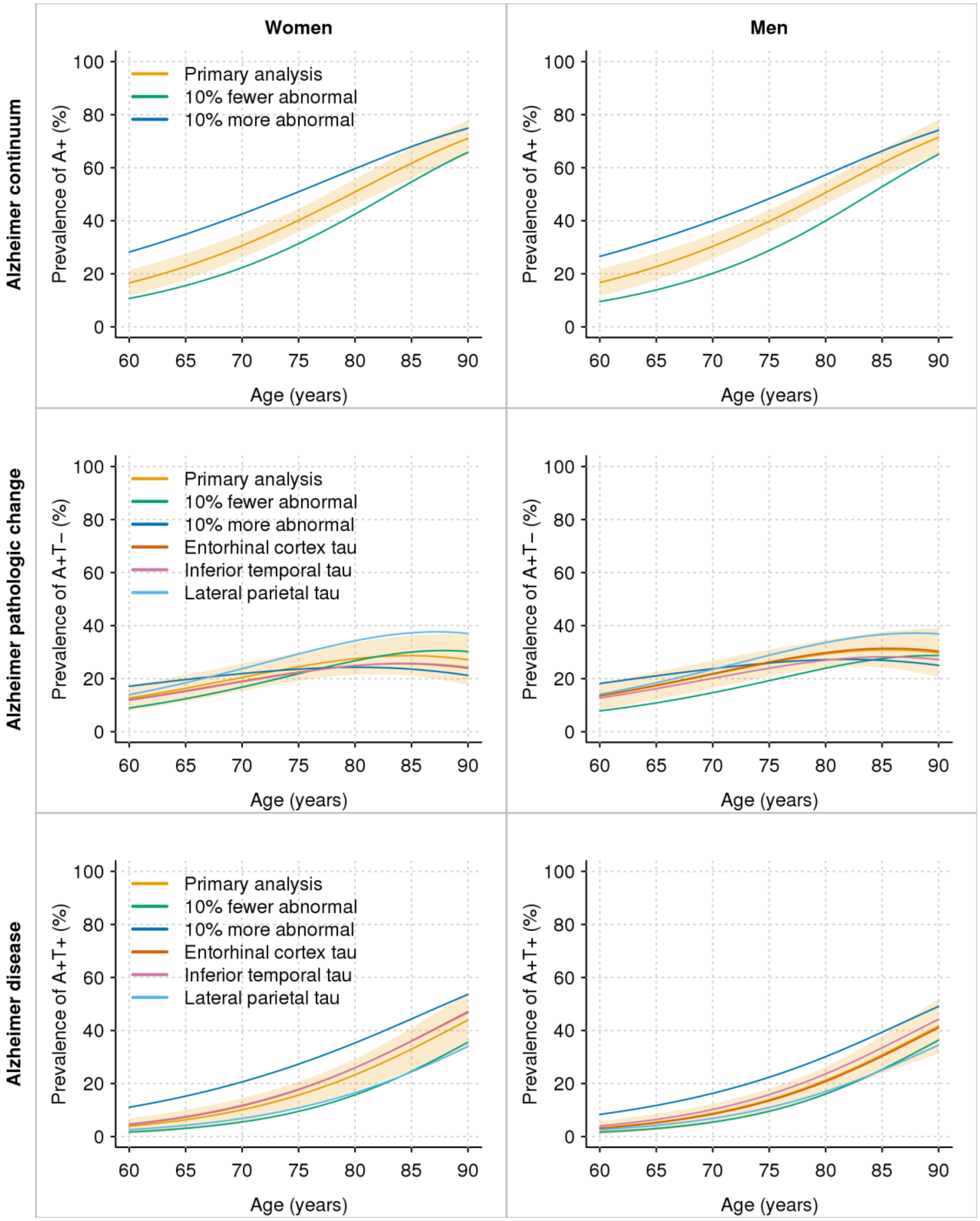

Estimated prevalence of Alzheimer continuum (A+), Alzheimer pathologic change (A+T−), and Alzheimer disease (A+T+) by age and sex. The primary analysis estimates with 95% pointwise confidence intervals based on jackknife methods are shown for reference. Estimates from sensitivity models include: varying the tau and amyloid cut-points to classify 10% fewer individuals as abnormal,

© 2019 Jack CR Jr et al. *JAMA Neurology*.

varying the tau and amyloid cut-points to classify 10% more individuals as abnormal, using entorhinal cortex (ERC) tau instead of the meta-ROI as the tau reporter ROI, using inferior temporal tau instead of the meta-ROI as the tau reporter ROI, and using lateral parietal tau instead of the meta-ROI as the tau reporter ROI. Inverse probability weights were used to account for potential participation bias related to age, sex, and education.

© 2019 Jack CR Jr et al. *JAMA Neurology*.

References

1. Roberts RO, Knopman DS, Syrjanen JA, et al. Weighting and standardization of frequencies to determine prevalence of AD imaging biomarkers. *Neurology*. 2017;89(20):2039-2048.
2. Jack CR, Wiste HJ, Weigand SD, et al. Defining imaging biomarker cut points for brain aging and Alzheimer's disease. *Alzheimers Dement.* 2017;13(3):205-216.

© 2019 Jack CR Jr et al. *JAMA Neurology*.
